# Exploring the Impact of the Synthesis Variables Involved in the Polyurethane Aerogels-like Materials Design

**DOI:** 10.3390/gels10030209

**Published:** 2024-03-20

**Authors:** Esther Pinilla-Peñalver, Darío Cantero, Amaya Romero, Luz Sánchez-Silva

**Affiliations:** Department of Chemical Engineering, University of Castilla-La Mancha, Avda. Camilo José Cela 12, 13071 Ciudad Real, Spain; esther.pinilla@uclm.es (E.P.-P.); dario.cantero@uclm.es (D.C.); amaya.romero@uclm.es (A.R.)

**Keywords:** polymer aerogel, chain extender, solids content, thermal conductivity, density, pilot scale

## Abstract

This research presents a novel approach to synthesising polyurethane (PUR)-based aerogels at the pilot scale, optimizing synthesis variables such as the gelation solvent, solids content, chain extender/isocyanate ratio, and dispersion mode. The solids content (2–11 wt.%) is the parameter with the most influence on the density of the aerogels, with a clear decrease in this property as the solids content decreases. On the other hand, it was demonstrated that minimizing the excess of ethylenediamine (used as chain extender) in relation to the isocyanate is a valuable consideration to improve the thermal conductivity of the aerogel. Related to the chain extender/isocyanate ratio, a compromise situation where the initial isocyanate reacts almost completely is crucial. Fourier-transform infrared spectroscopy was used to conduct such monitoring during the reaction. Once the conditions were optimised, the aerogel showing improved properties was synthesised using ethyl acetate as the gelling solvent, a 3.7 wt.% solids content, an ethylenediamine/isocyanate ratio of 0.20, and sonication as the dispersion mode, attaining a thermal conductivity of 0.030 W m^−1^ K^−1^ and a density of 0.046 g cm^−3^. Therefore, the synthesized aerogel emerges as a promising candidate for use in the construction and automotive industries.

## 1. Introduction

The current energy situation has promoted the development of different insulating materials for the building sector as one of the main focal points to achieve even greater energy savings. This helps to reduce energy consumption in situations with extreme temperatures and greenhouse gas emissions [1,2]. In this sense, aerogels are presented as an improved alternative to conventional insulating materials used in the construction industry, presenting much lower thermal conductivity values when compared to classic materials such as polyurethane foams. In addition, aerogels are also characterised by their low density, being ultra-light materials that do not compromise the structural integrity of the buildings in which they are to be installed [3,4,5], and present promising physicochemical properties that allow them to be used in a wide range of applications (thermal and acoustic insulation, catalysts, aerospace applications, among others) [6,7,8,9,10,11].

There are four main types of aerogels: organic, inorganic, carbon, and hybrid [12,13,14,15,16,17]. Each of these aerogels exhibits distinct properties, rendering them highly suitable for specific industrial applications. Within the organic group, polyurethane-based aerogels have been widely studied in recent years, synthesizing materials with very low thermal conductivities and densities [18]. These aerogels are mainly composed of polyurethane polymeric chains [19,20].

Polyurethane-based (PUR) aerogels were initially discovered in the late 1990s [3], exhibiting impressive results in terms of density and thermal conductivity. Subsequently, researchers achieved successful synthesis of polyurethane-polyisocyanurate aerogels characterized by low density and thermal conductivity values, followed by an in-depth investigation of these materials [18]. Subsequently, Rigacci et al. [19] investigated various approaches to enhance both the insulating and mechanical properties of the materials. This was achieved by combining two polyols with different functionalities and carefully adjusting the solubility of the reaction media. Notably, particular emphasis was placed on evaluating the impact of solubility on the thermal conductivity of the aerogels. Regarding the catalyst used for the synthesis, studies were conducted to investigate the influence of the catalyst concentration in the polyurethane synthesis process on the properties of the resulting aerogel [20]. The effect of the isocyanate and the polyol used for the synthesis was then examined by Chidambareswarapattar et al. [21], who studied different isocyanate and polyol combinations and ranged the formed PUR aerogels depending on their degree of flexibility, establishing a relationship between the structure and properties of the final aerogel and the functional group density of the monomer. More recently, Zhu et al. [12] studied the influence of the gelation solvent on the density, microstructure, and thermal conductivity of these materials, finding that the selected solvent has a significant impact on the aerogel properties.

PUR aerogels are usually synthesized by the acetone method [22,23], which is a particular type of sol–gel method in which the polymer (polyurethane) is generated in a multi-step synthesis process using acetone as a solvent. However, recent studies have shown that aerogels synthesized following this method degrade very rapidly after being synthesized [12]. To solve this problem, acetone can be replaced by other solvents (pure or in different volumetric mixtures) with similar Hansen solubility parameters to acetone to avoid undesired interactions during the reaction. Furthermore, it is crucial for the boiling temperatures of these alternative solvents to be slightly higher than that of acetone (56 °C). This ensures that they do not evaporate prematurely upon addition to the reaction mixture [24,25,26].

Another parameter to be considered when performing the polymerisation step is the proportion of chain extender added depending on the amount of free isocyanate groups at that time [27,28]. The chain extender compound should be added in a slight excess to promote the reaction with the isocyanate groups. However, it is important to avoid adding an excessive amount to prevent degradation over time, which could result in the loss of the distinctive physical and chemical properties that characterize these materials [27]. This careful balance ensures their suitability for future applications and prevents their wastage [29,30].

The final key variable in the polyurethane-in-water dispersion process is the method of agitation employed to achieve it. Mechanical agitation is commonly utilized by many researchers to ensure effective dispersion. This type of agitation is also employed throughout the stepwise polymerization process to facilitate the formation of the polyurethane material. However, several studies have shown that agitation by sonication is more efficient than classic mechanical agitation, resulting in better-dispersed hydrogels and improved characteristics of the final aerogels.

Furthermore, there is a notable absence of studies on the pilot-scale production of polyurethane aerogels. This gap in research is crucial, as the ability to scale up synthesis processes is imperative for the practical application of these materials in various industries. The successful translation to larger-scale production is essential for creating marketable materials with real-world applications. This study focused on conducting pilot-scale synthesis of polyurethane aerogels using modified recipes, which deviated from the conventional acetone method. The aim was to investigate the influence of these modifications on the properties of the produced aerogels, introducing a novel approach to the field. To achieve this, the freeze-drying method was selected as the drying technique, facilitating the removal of water from the initial hydrogel and enabling the formation of the desired porous structure in the aerogels. Thanks to this drying method, it was possible to synthesize structurally stable aerogels with high thermal insulating capacities while reducing energy consumption when compared to other drying techniques, such as supercritical drying [5,31,32], and maintaining an environmentally friendly synthesis route for these materials. Different characterization techniques were used to assess the physicochemical and morphological characteristics of the synthesized aerogels, such as the thermal conductivity and density, mechanical behaviour, thermal and dimensional stability, and porous structure. All these parameters were evaluated for aerogel samples resulting from changes in the gelling solvent, solids and chain extender content, and agitation method. Based on these outstanding characteristics, the selected aerogel sample holds great potential for various applications ranging from construction and building industries to even automotive applications.

## 2. Results and Discussion

### 2.1. Influence of the Synthesis Variables Involved in the Polyurethane Aerogel Design

The raw synthesis process for waterborne polyurethane (WBPUR) aerogels is described in the Materials and Methods section [33]. The impacts of key synthesis variables on the physicochemical properties of the materials were extensively assessed. These variables include the organic solvent nature, solids content, chain extender/isocyanate ratio, and polymer dispersion mode. Table 1 summarizes the experiments conducted to evaluate how each variable affects the hydrogel synthesis procedure (prior to the drying process), thus influencing the final physicochemical properties of the aerogels.

WBPUR aerogels were exhaustively characterized by different physicochemical techniques to evaluate their properties after the evaluation of the main key parameters of the synthesis process. The hydrophilic character of the samples was assessed by means of contact angle measurements, obtaining an average value of 84 ± 5° (*n* = 5) (see Figure S1, which exemplifies one of the samples obtained from experiment 12). All characterization techniques employed to evaluate the physicochemical properties of WBPUR aerogels are summarized in the ESM.S1.

#### 2.1.1. Influence of the Nature of the Organic Solvent

The influence of the nature of the organic solvent on the WBPUR aerogels’ properties was evaluated by adding different solvents (or their mixtures) to the reaction mixture prior to gel formation and the freeze-drying process (Table 1, set 1). Our previous experience using the acetone method [33] showed that it was difficult to work with this solvent due to its low boiling point in relation to the operating conditions used during the polyurethane synthesis. Thus, acetone was directly substituted by some other solvents with similar solubility parameters. ACN and EtOAc may be good candidates, as they meet these criteria. They are chemically inert, miscible with water, and have relatively low but slightly higher boiling points, which would overcome the limitations of acetone. The Hansen solubility parameters (δ_Dispersion_, δ_Polarity_, and δ_H-bonding_) included in Table S1 [34] are also very similar to those of acetone. Specifically, five proportions were tested, both pure and ACN:EtOAc mixtures of 25:75, 50:50, and 75:25 (*v*:*v*).

**Figure 1:**
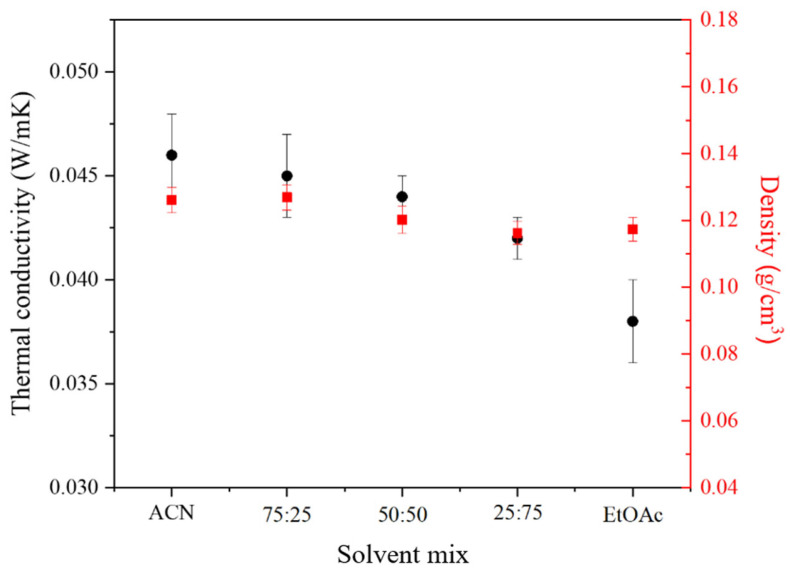
Influence of the organic solvent on the density and thermal conductivity of WBPUR aerogels (synthesis conditions: 10.8 wt.% solids content).

Figure 1 summarizes the results obtained in terms of density and thermal conductivity for the aerogels. Regardless of the organic solvent employed, the density of the aerogels remains almost constant at around 0.12 g cm^−3^ (similar δ_Dispersion_ values of solvents between 15.3 and 15.8 MPa^1/2^). Thus, density should not be considered a critical parameter when choosing between the different organic solvents used. However, the thermal conductivity of the materials is slightly dependent on this factor. While pure ACN-based aerogels display a thermal conductivity of 0.046 ± 0.002 W m^−1^ K^−1^, those of pure EtOAc-based ones exhibit a minimum of 0.038 ± 0.002 W m^−1^ K^−1^, showing a downward trend as the proportion of EtOAc increases. Accordingly, an increase in the δ_H-bonding_ parameter (higher EtOAc proportion) results in a better insulation capacity of the produced aerogels. This trend was also previously observed by Zhu et al. [12].

**Figure 2:**
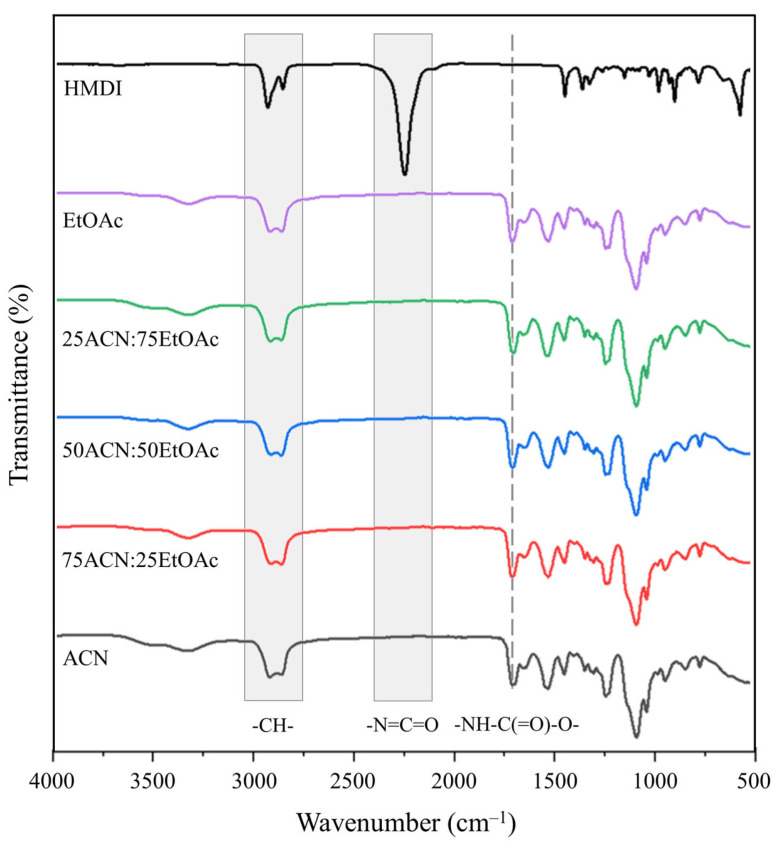
FT-IR spectra of pure isocyanate and WBPUR aerogels prepared using different gelation solvents.

The FT-IR spectra of the HMDI and synthesised WBPUR aerogels are shown in Figure 2. Based on observations, it appears that the chemical structure of the WBPUR aerogels is not affected by the nature of the solvent. All solvents tested exhibited similar profiles, including bands in the regions 3040–2760 cm^−1^ for the C-H stretching vibration of alkanes and 1710 cm^−1^ for the stretching vibration of urethane and allophanate groups. Additionally, the absence of an absorption band in the 2470–2025 cm^−1^ region indicates that there are no -N=C=O free groups present and that they have completely reacted to form urethane groups. In view of these results, EtOAc was selected as the organic solvent to prepare WBPUR aerogels with the improved properties.

In addition, the mechanical properties of the aerogel samples were investigated. Stress–strain profiles corresponding to the samples prepared with pure EtOAc and ACN as organic solvents were carried out. Young’s modulus values found for EtOAc and ACN were 0.195 ± 0.004 and 0.101 ± 0.003 MPa (*n* = 3), respectively. Samples prepared with mixtures of these solvents showed Young’s modulus values between these two values.

Based on the obtained results, it can be concluded that there are no significant differences in the density values of the samples, irrespective of the solvent used. However, the aerogels prepared using EtOAc as the organic solvent were selected due to their slightly higher mechanical strength and, primarily, their lower thermal conductivity.

#### 2.1.2. Influence of Solids Content

The effect of the solids content used in WBPUR aerogel synthesis prepared with pure EtOAc was evaluated from 3.2 to 10.8 wt.% (Table 1, sets 2 and 4). The solids content was calculated using Equation (1):

(1) $$\mathrm{Solids}\;\mathrm{content}\;\left(\mathrm{wt}.\;\%\right)=\frac{\sum_{\mathrm{weight}}(\mathrm{HMDI}+\mathrm{PEG}+\mathrm{DMPA}+\mathrm{TEA}+\mathrm{EDA})}{\sum_{\mathrm{weight}}(\mathrm{HMDI}+\mathrm{PEG}+\mathrm{DMPA}+\mathrm{TEA}+\mathrm{EDA}+\mathrm{water})}\cdot 100$$

**Figure 3:**
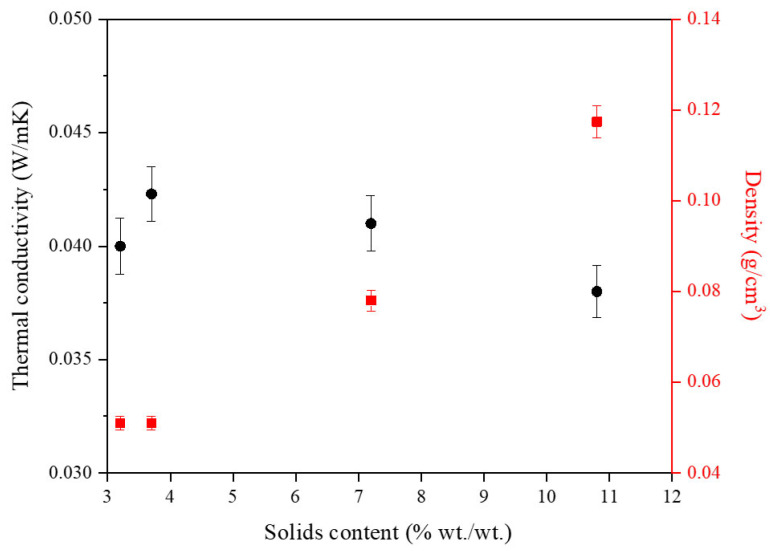
Influence of the solids content on the density and thermal conductivity of WBPUR aerogels.

As can be observed in Figure 3, there is a clear relationship between the solids content and the density of the aerogel samples. Samples with a higher solids content exhibit higher density values than those with a lower solids content. The high lightness of these type of samples can be appreciated in Figure S2, where a common plant leaf is able to support the aerogel sample without breaking. This effect is such that, physically, samples with less than a 3.7 wt.% solids content are so light that they deform very easily to the point where it becomes noticeable. Figure S3 illustrates the visual appearance of an aerogel with a solids content of 3.2 wt.%. The image clearly shows the deformation that occurs when the aerogel meets any material, leaving a distinct mark on its surface. Furthermore, due to the low solids content, the sample is unable to regain its original shape when subjected to minor deformations. The impact of the solids content on the thermal conductivities of WBPUR aerogels is also included in Figure 3. It can be observed that all thermal conductivity values are quite similar, with average measurements ranging from 0.038 to 0.041 W m^−1^ K^−1^. However, a slight decrease in conductivity can be noticed as the solids content increases.

**Figure 4:**
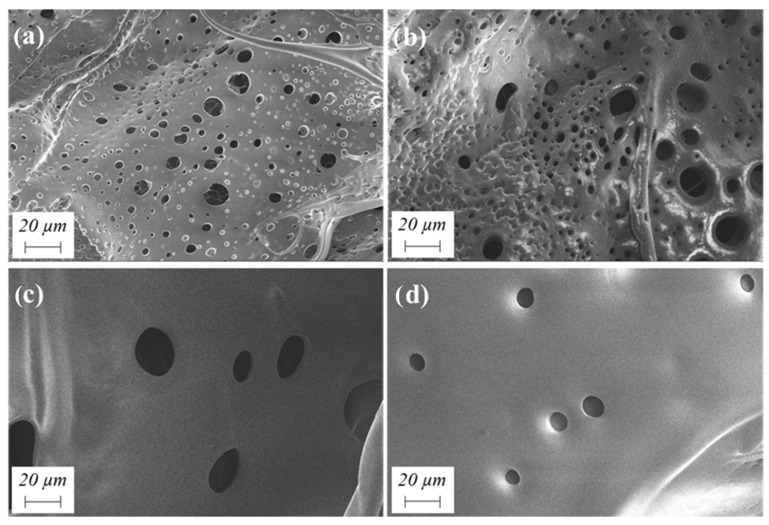
SEM micrographs of WBPUR aerogels prepared using EtOAc solvent with (**a**) 3.2, (**b**) 3.7, (**c**) 7.2, and (**d**) 10.8 wt.% solids content.

The high-resolution scanning electron microscopy (HRSEM) images presented in Figure 4 provide visual evidence of the porous structures of the aerogel [35,36]. Aerogels prepared with lower solids contents (Figure 4a,b) exhibit a primarily mesoporous structure, which is characteristic of aerogels. In contrast, aerogels with higher solids contents (Figure 4c,d) display structures with predominantly macroporous characteristics, although denser than the previous ones. Therefore, the SEM micrographs corroborate the findings obtained earlier using the density scanner.

**Figure 5:**
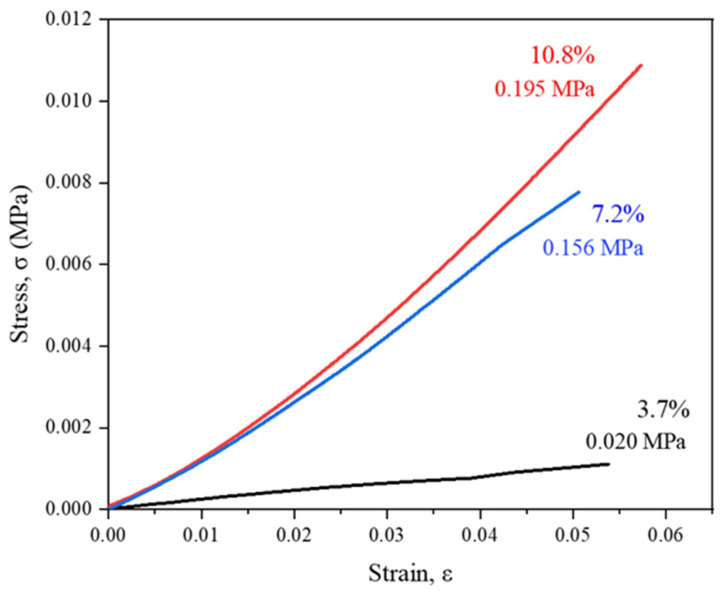
Compressive stress–strain curves of WBPUR aerogel prepared with different solids contents.

The solids content’s influence on the mechanical properties of the obtained aerogels was also evaluated. The elastic area of the stress–strain curves (lineal trend) is represented in Figure 5. This graph displays the Young’s modulus values alongside the corresponding data. It is evident that there is a decrease in Young’s modulus values as the solids content in the sample decreases. Notably, a nearly 10-fold decrease in Young’s modulus is observed for solids contents ranging from 10.8% to 3.7% by weight. Therefore, aerogels prepared with higher solids contents exhibit enhanced mechanical strength, characterized by increased stiffness, compared to those with lower contents. It should be noted that the sample with the lowest solids content, 3.2% by weight, is extremely deformable, making it challenging to obtain a reliable Young’s modulus for this analysis. Thus, no specific Young’s modulus value is provided for this sample due to the difficulties in sample preparation for this type of analysis.

Considering the obtained results, aerogels prepared using 3.7 wt.% were selected as the most favourable for their lower density values.

#### 2.1.3. Influence of Chain Extender/Isocyanate Ratio

**Figure 6:**
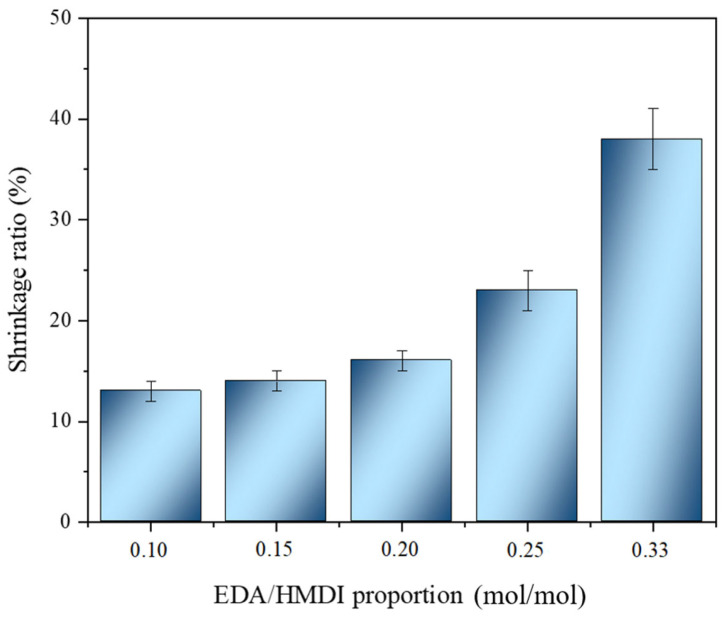
Shrinkage ratios corresponding to WBPUR aerogels obtained using different chain extender/isocyanate ratios (measured one month after their preparation).

**Figure 7:**
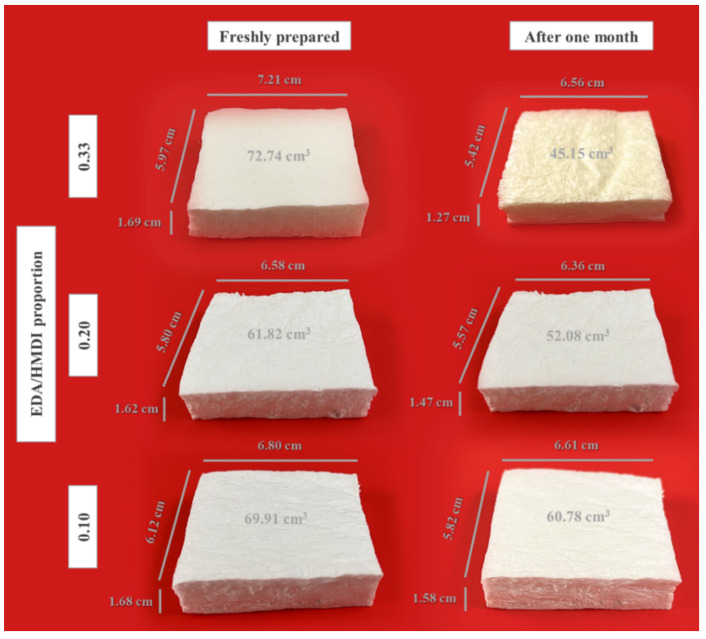
Physical appearance of WBPUR aerogels. WBPUR aerogels obtained using different chain extender/isocyanate ratios (freshly prepared and one month after their preparation).

The effect of the EDA content on the synthesis of WBPUR aerogels was evaluated (Table 1, sets 3 and 5). As previously mentioned, an excess of the chain extender is necessary when added to the prepolymer to ensure a complete reaction of the isocyanate (limiting reagent) and the absence of any free -N=C=O groups. However, a large excess can cause agglomeration during polymerization, hindering the process. Additionally, it has been observed that over time, the final product may develop a yellowish colour and increased stiffness, which is likely attributed to oxidation of the high amine content. Furthermore, the samples lose their dimensional stability, particularly in terms of thickness, as they reduce to thin films resembling a coating. Previous aerogels, which were prepared using acetone as the gelation solvent and an EDA/HMDI ratio of 0.33 [33], exhibited an average shrinkage ratio of 62 ± 6% (*n* = 3) 5 months after their preparation (see Figure S4). To overcome these limitations, it may be possible to reduce the amine content instead. Therefore, the EDA/HMDI ratio was evaluated for aerogels ranging between 0.10 and 0.33. The shrinkage ratios presented in Figure 6 were measured for samples placed on a horizontal support, and the reported values represent the average results from triplicate samples. It can be appreciated (Figure 7) that as the mentioned ratio decreased, more stable aerogels were obtained in terms of dimensionality (less shrinkage) and visual appearance (less change in colour due to degradation), obtaining fewer stable aerogels when a ratio of 0.33 is used. This fact could be attributed to the oxidation of free amines, which are found in excess in the reaction and have no isocyanate groups to react with, when samples are in contact with air. However, there comes a point where even if the studied ratio is reduced, the shrinkage of the samples is almost constant. Thus, ratios ranging between 0.20 and 0.10 imply a higher level of dimensional stability for the materials, which is crucial for practical industrial applications, such as construction and building materials. Nevertheless, it has not been feasible to select one specific ratio from this range for further investigation because it is unclear which ratio achieves a complete isocyanate reaction without a highly excessive presence of amines. Consequently, a thorough study of the chemical structure of the aerogels is necessary to make an informed decision.

**Figure 8:**
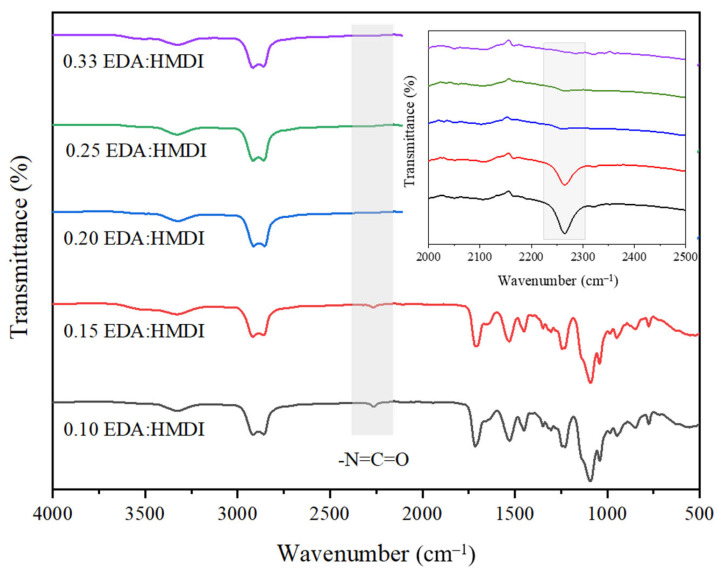
FT-IR profiles of the synthesized WBPUR aerogels obtained using different chain extender/isocyanate ratios. Inset: amplified region showing the -N=C=O groups.

Infrared spectroscopy was used to characterize the chemical structure of the final aerogels and to monitor the influence of this variable on the synthesis process. Figure 8 includes FT-IR profiles of samples synthesizing using different EDA/HMDI ratios. It can be observed that as the ratio decreased from 0.33 to 0.15, there is a slight presence of free -N=C=O functionalities. This observation becomes more pronounced for smaller ratios such as 0.10, as expected. In other words, as the amount of chain extender decreases, the number of unreacted isocyanate groups increases. To ensure the absence of free isocyanate groups in the aerogel material, a ratio of 0.20 was selected for further studies. This specific ratio was selected because it represents the minimum ratio at which no free isocyanate groups were detected, indicating their complete reaction. These results further support the previously described good dimensional stability of the aerogels, highlighting the successful formation of the desired chemical structure and the absence of unreacted isocyanate groups that could potentially affect the performance or stability of the aerogels. Hence, while the chemical composition of the polymer remains intact, its long-term stability is compromised when subjected to high EDA:HMDI ratios.

**Figure 9:**
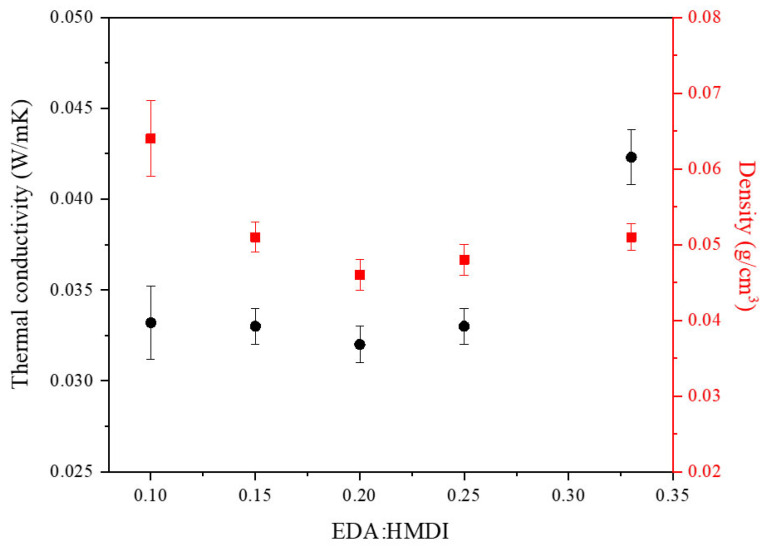
Influence of EDA/HMDI ratio in the density and thermal conductivity of WBPUR aerogels.

The variable under consideration also influenced other physical properties, including thermal conductivity and density. The trends in these properties are depicted in Figure 9. The thermal conductivity values remained practically constant up to an EDA/HMDI ratio value of 0.25. From that value, the conductivity began to increase more significantly. On the other hand, the density values decreased with the EDA/HMDI ratio until a value of 0.20, after which the density of the samples remains practically unchanged.

**Figure 10:**
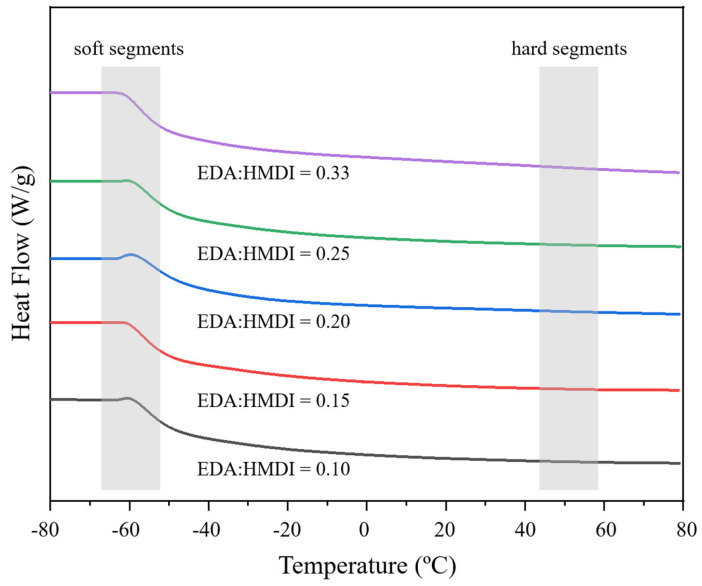
DSC thermograms of the synthesized WBPUR aerogels obtained using different chain extender/isocyanate ratios.

In addition, the crystalline phases of WBPUR aerogels were assessed by differential scanning calorimetry (DSC). A PUR structure is formed by the hard (OH groups from polyol) and soft segments (NCO groups from the isocyanate and chain extender). As can be observed in Figure 10, the heating profiles show a significant slope attributed to the soft segments (−58 °C), while that corresponding to the hard segments is not as appreciated [37,38]. As expected, the influence of the EDA/HMDI ratio does not affect the heating profiles.

#### 2.1.4. Influence of Polymer Dispersion Mode

**Figure 11:**
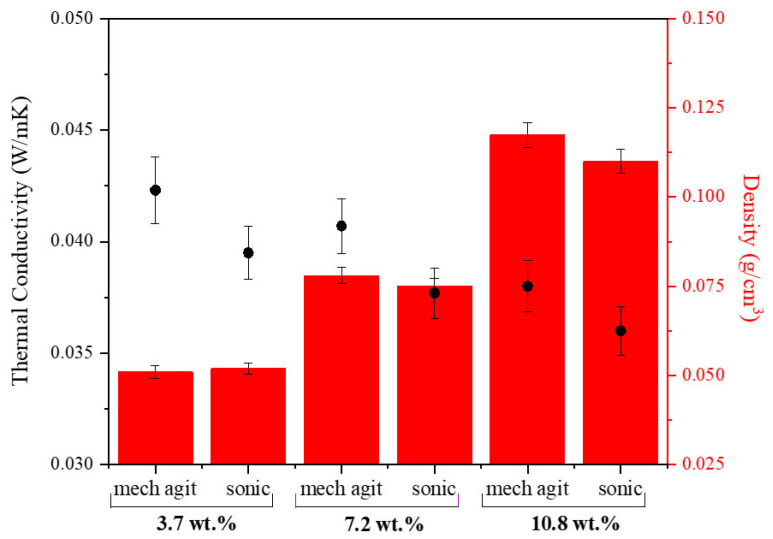
Thermal conductivity and density values found for the synthesized aerogels with different solids contents and dispersion modes (synthesis conditions: 0.33 EDA/HMDI ratio).

**Figure 12:**
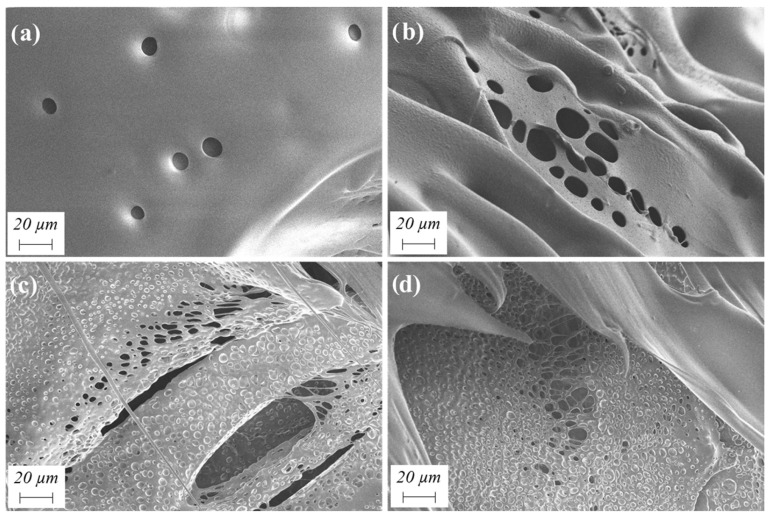
SEM micrographs corresponding to the obtained WBPUR aerogels using (**a**,**b**) mechanical agitation and (**c**,**d**) sonication as dispersion mode (synthesis conditions: 10.8 wt.% solids content and 0.33 EDA/HMDI ratio).

After polymer formation, it needs to be dispersed in water. Two methods of dispersion have been investigated to attain a higher level of uniformity in the mixture in the shortest possible time. Up until now, all experiments were carried out using mechanical agitation at 900 rpm. Moreover, sonication using a H40 sonotrode was also used as an alternative dispersion mode. This influence was checked for different solids contents (3.2–10.8 wt.%) and a 0.33 EDA/HMDI ratio (Table 1, sets 2 and 4). Interestingly, when aerogels with higher solids contents (7.2–10.8 wt.%) are prepared, incorporating sonication during the polymer dispersion stage results in lower thermal conductivities and slightly lower densities in the final aerogels (Figure 11). SEM micrographs reveal that the sonication mode attempts to create a more porous structure compared to mechanical agitation. However, it is noteworthy that a significant portion of these pores do not open, even with prolonged dispersion times (Figure 12). On the other hand, for lower solids content ranges (<7.2 wt.%), the choice of dispersion mode does not impact the density of the samples. Mechanical agitation was found to provide sufficient energy for dispersing low solids contents. Nevertheless, using sonication did lead to an improvement in the insulating capacities of the aerogels (Figure 11).

This influence was checked for different EDA/HMDI ratios, with the solids content remaining constant at 3.7 wt.%, specifically for that previously selected and for those closest to them (0.15–0.33) (Table 1, sets 3 and 5). Based on the obtained results, it is apparent that mechanical agitation is slightly more time efficient during the dispersion stage, and the density properties of the aerogels were found to be very similar for the different dispersion methods. Furthermore, it was confirmed that the use of sonication as a dispersion method leads to a reduction in thermal conductivity values, like the observations made between sets 2 and 4. Therefore, mainly depending on the solids content of the sample and of the property to be improved, one method of dispersion is of more interest than the other.

**Figure 13:**
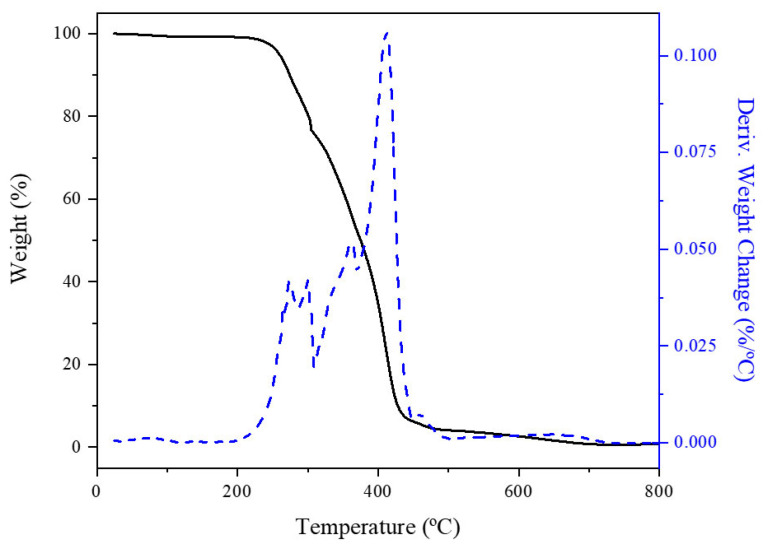
TGA (solid line) and DTGA (dotted line) curves of WBPUR aerogel (synthesis conditions: EtOAc, 3.7 wt.% solids content and 0.2 EDA/HMDI ratio).

Thermogravimetric analyses (TGA) carried out under an N_2_ stream were performed to evaluate the thermal stability of the WBPUR aerogels. The same profiles were found for all samples regardless of the influence analysed (nature of the organic solvent, the solids content, or the proportion of the chain extender). For clarification, Figure 13 corresponds to the results for one of the samples obtained from experiment 12 (Table 1), matching the type of aerogel considered to be the most suitable for the reasons discussed above. It can be appreciated the thermal decomposition process combined with the derivative of the weight versus the temperature to which the sample is subjected. Different stages of decomposition are shown in the TGA curve [39]. the aerogel samples are thermically stable up to 230 °C, the temperature at which they have lost any trace of water they may contain and other residual solvents such as the corresponding organic solvent and NMP. Subsequently, there is another stage between 230 and 440 °C that implies the highest amount of mass loss (>90 wt.%) and is related to the decomposition of the networks of polyurethane. Finally, from 440 to 700 °C, another stage is observed which can be assigned to the decomposition of ester groups [40]. Around 1 wt.% remained at the end of this stage.

## 3. Conclusions

WBPUR aerogels were designed at a pilot scale via a polymerization process and freeze-drying technique, using different organic solvents, solids contents, amines contents, and water dispersion modes. These crucial factors have demonstrated significant influence on the physicochemical properties of the resulting aerogel materials. EtOAc proved to be a good alternative candidate to acetone as the gelling solvent considering their similar solubility parameters and higher boiling temperature. As the solids content in the aerogel samples decreases, it implies low-density materials; however, a very low amount gives rise to materials so light that they deform. SEM micrographs corroborate the results with an increase in sample porosity. An excess of amines in the aerogel samples from the chain extender influences their degradation over time in terms of dimensional stability, stiffness and colouring of the sample. Although a lower EDA/HMDI ratio results in lower thermal conductivities, 0.20 was selected as the minimum amount for the isocyanate to fully react (no free -N=C=O functionalities) according to the FT-IR results. Finally, the type of agitation for polymer dispersion was shown to affect the density and thermal conductivity of the samples when using sonication versus mechanical agitation; however, this only affected high solids contents in the samples. In conclusion, the exceptional lightness of aerogels, coupled with their insulating and structural properties, presents promising prospects for innovative applications in various sectors, such as construction or the automotive industry. Compared to conventional materials, aerogels offer versatile and sustainable solutions with the potential for significant advancements.

## 4. Materials and Methods

### 4.1. Chemicals

The chemicals used are listed below: polyethylene glycol [PEG, number-average molecular weight (Mn) = 2000 g·mol^−1^, Sigma-Aldrich, St. Louis, MO, USA]; 2,2-bis(hydroxymethyl)propionic acid [DMPA, 98%; Aldrich Chemicals, Milwaukee, WI, USA]; 1-methyl-2-pyrrolidinone anhydrous [NMP, 99.5%, Sigma-Aldrich)]; dibutyltin dilaurate [DBTDL, 95%, Aldrich Chemicals]; 4,4′-methylenebis(cyclohexyl isocyanate) [HMDI, 90% mixture of isomers, Sigma-Aldrich]; ethylenediamine [EDA, ≥99%, Sigma-Aldrich]; triethylamine [TEA, ≥99%, Sigma-Aldrich]; acetonitrile anhydrous [ACN, 99.8%, Sigma-Aldrich]; and ethyl acetate anhydrous [EtOAc, 99.8%, PanReac, Barcelona, Spain]. Water was purified by distillation followed by deionization using ion-exchange resins. PEG and DMPA were dried overnight at 60 °C under vacuum before use to remove residual water. Other chemicals were used as purchased, unless otherwise stated.

### 4.2. Synthesis Procedure of Waterborne Polyurethane Aerogels

**Figure 14:**
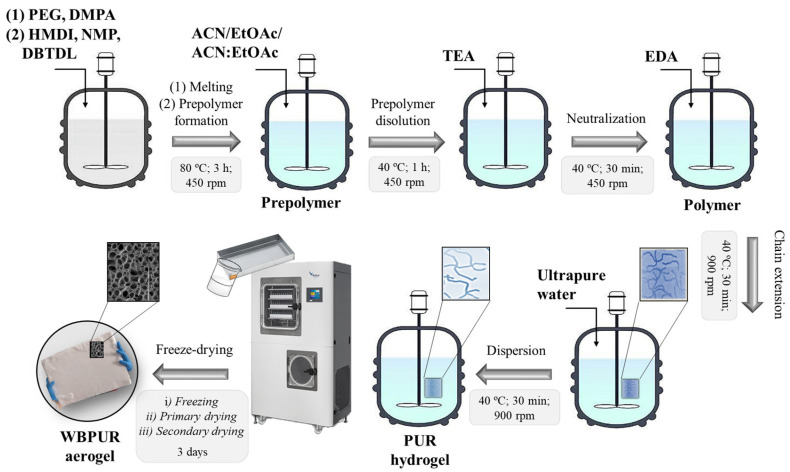
Schematic process of WBPUR aerogel design via stepwise polymerization and freeze-drying methods.

The synthesis process of WBPUR aerogels involved a slightly modified polymerization method followed by a freeze-drying process, as described in previous studies [33]. The notable variation in the procedure was the utilization of various organic solvents, including EtOAc, among others. Figure 14 and Figure S5 schematically summarise the stepwise polymerization process followed.

Initially, PEG (polyol, 0.06 mol) and DMPA (emulsifier agent, 0.17 mol), previously conditioned for its dehydration, are melted under vigorous mechanical stirring in a jacketed vessel at 80 °C. Once the mixture is melted, HMDI (isocyanate, 0.34 mol), NMP (anhydrous solvent, 0.17 mol), and DBTDL (catalyst, 8.86·10^−4^ mol) are added and allowed to react for 3 h at 450 rpm while maintaining the same temperature. After this step, the temperature is lowered to 40 °C before adding the required organic solvent (ACN, EtOAc, or a mixture of both). The mixture is left to stir for a further hour before adding TEA (neutralizer agent, 0.20 mol) to neutralize the DMPA acidic functionalities. This step takes 30 min to reach the complete neutralization of carboxylic groups. All previous steps were carried out in a stream of N_2_ to create an inert atmosphere. The next step consists of the extension of polymers chains, for which the corresponding EDA amount (chain extender, 0.030–0.045 mol) is used in slight excess, adding another 30 min to the process. Thus, an EDA/HMDI ratio was evaluated in the 0.10–0.33 range. To calculate this ratio, the HMDI content is considered just prior to the chain extender stage (prior to EDA addition) and not at the beginning of the process [41]. The final stage of the synthesis method is reached by adding the corresponding water content and complete polymer dispersion either by mechanical (900 rpm) or sonication agitation (H40 sonotrode (24 kHz, 400 W, 50% amplitude)) for ca. 30 min. The added water content will affect the solids contents of the samples (tested between 10.8 and 3.2 wt.%).

Once the mixture is entirely dispersed in water, it is transferred to steel trays (43.5 cm of length × 34 cm of width and a thickness of 2 cm). The synthesis procedure allows the production of aerogel panels with a surface area of 0.9 m^2^ and a thickness of 1.5–2 cm, which are obtained after undergoing a freeze-drying cycle with different stages (freezing, primary and secondary drying). During drying, water is removed from the samples by sublimation, generating the final aerogel. Specifically, the aerogel formation process involved freezing for 3 h at −40 °C, followed by a primary drying phase lasting 60 h at 25 °C and 200 µbar. Lastly, a secondary drying step was performed for 5 h at 40 °C. During the freeze-drying process, the cooling or heating ramps to reach the desired temperature were set to last for 1 h.

### 4.3. Characterization Techniques of Waterborne Polyurethane Aerogels

The physicochemical properties of the synthesized aerogels were measured using the characterization techniques described below. In addition, more details about how the measurements are carried out for each technique are provided in the ESM.S1.

#### 4.3.1. Density

The density (ρ, g cm^−^^3^) was obtained using the ratio between the mass of the aerogels and their geometrical volume, as described in ASTM D1622/D1622M-14 [42]. The density of the materials was more accurately determined using a 3D scanner (REXCAN DS3 Silver, eQuality Tech Inc., Rochester Hills, MI, USA). The scanner is equipped with the ezScan programme for drawing the sample in 3D, while the Geometric Wrap programme is used for volume calculation.

#### 4.3.2. Thermal Conductivity

The thermal conductivity (λ, W m^−^^1^ K^−^^1^) of the aerogels was measured using a Heat Flow Meter (Tempos, Meter, Munich, Germany). The KS-3 sensor was inserted into the material, and the equipment measured the thermal conductivity of the given material using the insulation mode. In addition, the thermal conductivity of the materials was evaluated by heat transfer between two parallel plates (HFM 300, Linseis, Selb, Germany).

#### 4.3.3. Shrinkage Ratios

The aging shrinkage ratio (τ_f_, %) of each sample was calculated by relating the original volume of the freshly prepared aerogel to the final volume of the sample at the time considered since its preparation.

#### 4.3.4. Mechanical Characteristics

The Young’s modulus was determined by dynamic mechanical analysis (DMA, 1STARe System, Mettler Toledo, Madrid, Spain) of the WBPUR aerogels by means of stress–strain analysis in compression mode.

#### 4.3.5. Chemical Structure

The chemical structures of the samples were studied using an infrared spectrometer (Spectrum Two, PerkinElmer, Waltham, MA, USA) equipped with a universal attenuated total reflectance (ATR) accessory and PerkinElmer Spectrum V10.4.3 software for data acquisition.

#### 4.3.6. Morphological Structure

The morphological structure was analysed using a high-resolution scanning electron microscope (HRSEM, GeminiSEM 500, ZEISS, Jena, Germany) with an energy dispersive spectroscopy (EDS) sensor of 80 mm^2^ and another one of electron backscatter diffraction (EBDS).

#### 4.3.7. Thermogravimetric Analysis

Thermogravimetric analyses (TGA) were carried out to evaluate the thermal stability of the synthetized aerogels by using a thermal analyser (TGA, 2STARe system, Mettler Toledo, Madrid, Spain). Furthermore, the glass transition temperature (T_g_) was determined on a differential scanning calorimeter (DSC, 2STARe system, Mettler Toledo, Madrid, Spain). For TGA and DSC experiments, 2STARe V16.30 software was used for data acquisition.

#### 4.3.8. Contact Angle

Contact angles were measured using an optical tensiometer (Theta Lite, Biolin Scientific, Gothenburg, Sweden) at 25 °C.

## Figures and Tables

**Table 1:** Summary of the experiments carried out. Shaded boxes indicate the experiment selected in each set to progress to the subsequent evaluation (43.5 cm × 34 cm aerogel panels).

	Experiment	Organic Solvent Proportion	Solids Content (wt.%)	EDA/HMDI Ratio (mol/mol)	Dispersion Mode
Set 1	1	100EtOAc	10.8	0.33	Mechanical agitation
	2	25ACN:75EtOAc			
	3	50ACN:50EtOAc			
	4	75ACN:25EtOAc			
	5	100ACN			
Set 2	6	100EtOAc	10.8	0.33	Mechanical agitation
	7		7.2		
	8		3.7		
	9		3.2		
Set 3	10	100EtOAc	3.7	0.33	Mechanical agitation
	11			0.25	
	12			0.20	
	13			0.15	
	14			0.10	
Set 4	15	100EtOAc	10.8	0.33	Sonication
	16		7.2		
	17		3.7		
	18		3.2		
Set 5	19	100EtOAc	3.7	0.33	Sonication
	20			0.25	
	21			0.20	
	22			0.15	

EtOAc: ethyl acetate; ACN: acetonitrile; EDA: ethylenediamine; HMDI: 4,4′-methylenebis(cyclohexyl isocyanate).

## Data Availability

The original contributions presented in the study are included in the article, further inquiries can be directed to the corresponding authors.

## Supplementary Materials

The following supporting information can be downloaded at https://www.mdpi.com/article/10.3390/gels10030209/s1: Figure S1: Contact angle of WBPUR aerogel; Figure S2: Aerogel sample with 3.7 wt.% solids content held on a plant to demonstrate its lightness; Figure S3: Visual appearance of WBPUR aerogel containing 3.2 wt.% solids content after being subjected to different stresses; Figure S4: Illustration of WBPUR aerogels-based acetone solvent using 7.5 wt.% solids content and 0.33 EDA/HMDI ratio from their (A) front view and (B) profile view. On the right of each photograph is the freshly prepared sample and on the left is after 5 months of formulation; Figure S5: Schematic process of WBPUR aerogel synthesis. Table S1: Hansen solubility parameters of the organic solvents evaluated.
