# Supplementary material for: Exploring the Impact of the Synthesis Variables Involved in the Polyurethane Aerogels-like Materials Design
Source: Gels. 2024 Mar 20;10(3):209. doi: 10.3390/gels10030209 (PMC10970664; doi:10.3390/gels10030209)
Supplement: Supplementary file 1 [file gels-10-00209-s001.zip › gels-2914849-supplementary.pdf]

Supplementary Material

Exploring the impact of the synthesis variables involved in the polyurethane aerogels-like materials design

Department of Chemical Engineering, University of Castilla-La Mancha,
Avda. Camilo José Cela 12, 13071 Ciudad Real, Spain;
esther.pinilla@uclm.es (E.P.-P.); dario.cantero@uclm.es (D.C.);
amaya.romero@uclm.es (A.R.)
* Correspondence: marialuz.sanchez@uclm.es

S1. Experimental Section

Characterization of waterborne polyurethane aerogels

Density

The density (ρ, g cm^{-3}) was measured by dividing the mass of the obtained aerogels by their geometrical volume, as described in ASTM D1622/D1622M-14 [1]. The density of the materials was more accurately determined using a 3D scanner (REXCAN DS3 Silver, eQuality Tech Inc., Rochester Hills, Michigan, USA). The scanner is equipped with the ezScan programme for drawing the sample in 3D, while the Geometric Wrap programme is used for volume calculation.

For measurements, three square-shaped samples from the top, middle, and bottom zones of each synthesized aerogel were taken out and weighted to calculate the average density. The density of the samples was calculated when they were freshly synthesised.

Thermal conductivity

The thermal conductivity (λ, W m^{-1} K^{-1}) of the aerogels was measured using a Heat Flow Meter (Tempos, Meter, München, Germany). The KS-3 sensor was inserted into the material, and the equipment measures the thermal conductivity of the given material using the insulation mode. In addition, the thermal conductivity of materials was evaluated by heat transfer between two parallel plates (HFM 300, Linseis, Selb, Germany). For that, samples with dimensions of 30 x 30 cm are required. These analyses were conducted at five intermediate temperatures: (1) T_m: 0 ºC (T_i: -5 ºC, T_f: 5 ºC); (2) T_m: 10 ºC (T_i: 5 ºC, T_f: 15 ºC); (3) T_m: 20 ºC (T_i: 15 ºC, T_f: 25 ºC); (4) T_m: 30 ºC (T_i: 25 ºC, T_f: 35 ºC) and (5) T_m: 40 ºC (T_i: 35 ºC, T_f: 45 ºC).

Shrinkage ratios

The aging shrinkage ratio (τ_f) of each sample was calculated using the following equation 1, which relates the original volume of the freshly prepared aerogel (V_o) to the final volume of the sample at the time considered since its preparation (V_f):

$$\tau_f = 1 - (V_f/V_0) \cdot 100 \qquad \text{Equation (S1)}$$

Mechanical characteristics

The *Young's modulus* was determined using dynamic mechanical analysis (DMA, 1STARe System, Mettler Toledo, Madrid, Spain) of the polyurethane aerogels by means of stress-strain analysis in compression mode. Tests were performed on cubic samples of 10 x 10 x 6 mm in ambient conditions applying forces between 0 and 2 N.

Following the compression tests, the force (*F*) and displacement data were obtained. To plot the strain–stress curves, stress (σ) and longitudinal strain (ε) values are calculated thanks to the following equations 2 and 3, respectively:

$$\sigma = F/S_0 \qquad \text{Equation (S2)}$$

where:

F corresponds to the force (in Newtons, N) applied to the sample surface and S_0 is the initial cross section of the sample (in mm^2), calculated from its initial diameter.

$$\varepsilon = \Delta l(l_0 - l_1)/l_0 \qquad \text{Equation (S3)}$$

where:

l_0 and l_1 corresponds to the initial and the final length of the sample.

Chemical structure

The chemical structure of the samples was studied using an infrared spectrometer (Spectrum Two, PerkinElmer, Waltham, Massachusetts, USA) equipped with a universal attenuated total reflectance (ATR) accessory and PerkinElmer Spectrum V10.4.3 software for data acquisition. The spectra were collected by accumulating 64 scans in the range of 500-4000 cm^{-1} and using an optical resolution of 2 cm^{-1}.

Morphological structure

The *morphological structure* was analyzed using a high-resolution scanning electron microscopy (HRSEM, GeminiSEM 500, ZEISS, Jena, Germany) with an energy dispersive spectroscopy (EDS) sensor of 80 mm^2 and another one of electron backscatter diffraction (EBDS).

Thermogravimetric analysis

Thermogravimetric analyses (TGA) were carried out to evaluate the *thermal stability* of the synthetized aerogels by using a thermal analyser (TGA, 2STARe system, Mettler Toledo, Madrid, Spain). Furthermore, the glass transition temperature (T_g) was determined on a differential scanning calorimetry (DSC, 2STARe system, Mettler Toledo, Madrid, Spain) using a heating rate of 20 K min^{-1} from -80 to 80 ºC. For TGA and DSC experiments, 2STARe V16.30 software was used for data acquisition.

Contact angle

Contact angles were measured using an optical tensiometer (Theta Lite, Biolin Scientific, Gothenburg, Sweden) at 25 ºC. For that, two microliters of water were deposited onto the surface of polyurethane aerogels. The average contact angle was obtained by measuring it on three different drops on each sample and on each of the drops on their left and right sides.

Reference

[1] *ASTM D1622-08*; Standard Test Method for Apparent Density of Rigid Cellular Plastics. American Society for Testing and Materials (ASTM): West Conshohocken, PA, USA, 2020.

S2. Figures and tables

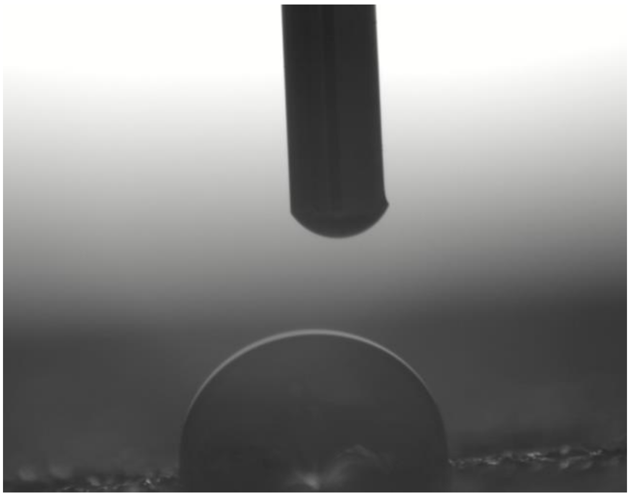

Figure S1 Contact angle of WBPUR aerogel.

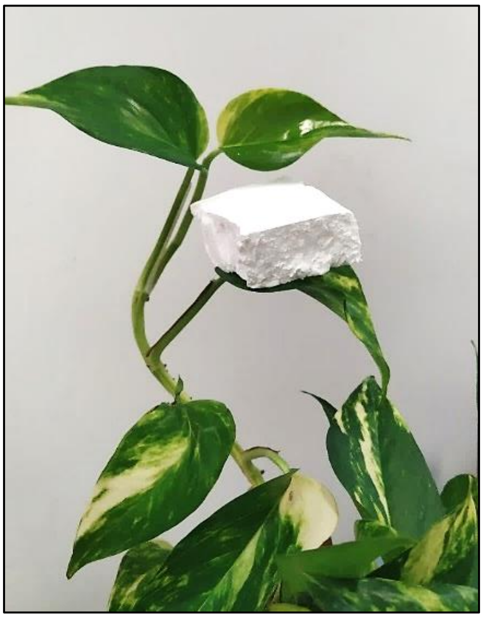

Figure S2 Aerogel sample with 3.7 wt.% solids content held on a plant to demonstrate its lightness.

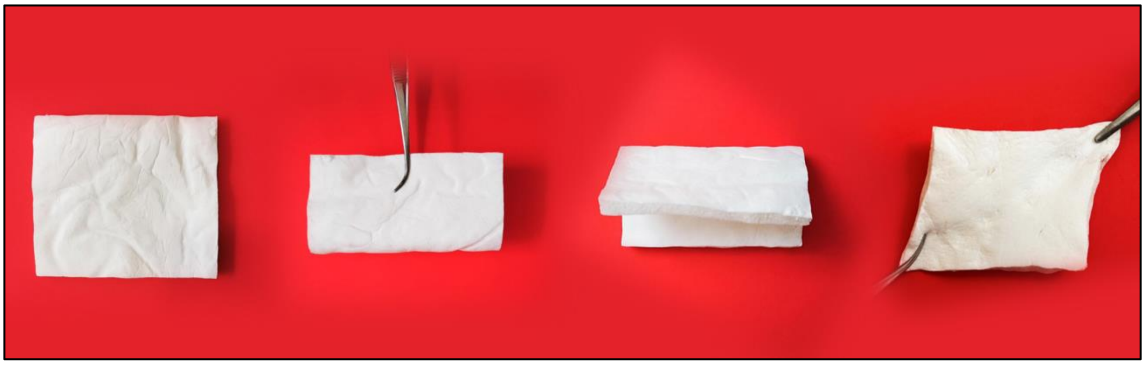

Figure S3 Visual appearance of WBPUR aerogel containing 3.2 wt.% solids content after being subjected to different stresses.

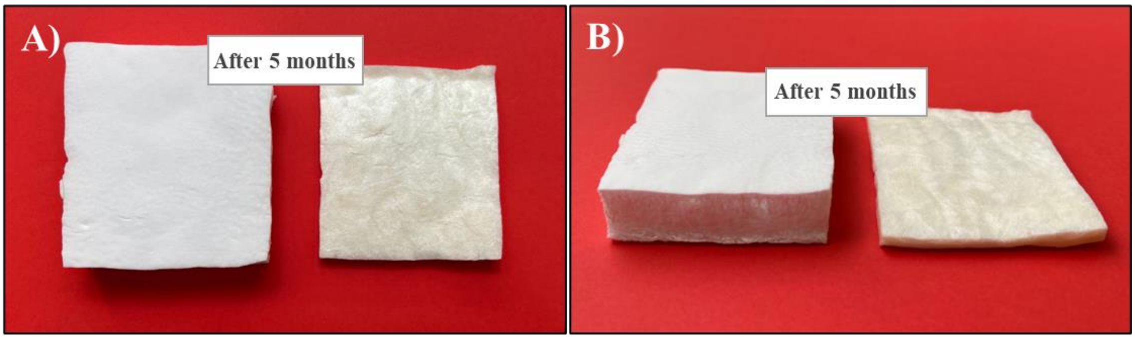

Figure S4 Illustration of WBPUR aerogels-based acetone solvent using 7.5 wt.% solids content and 0.33 EDA/HMDI ratio from their (A) front view and (B) profile view. On the right of each photograph is the freshly prepared sample and on the left after 5 months of formulation.

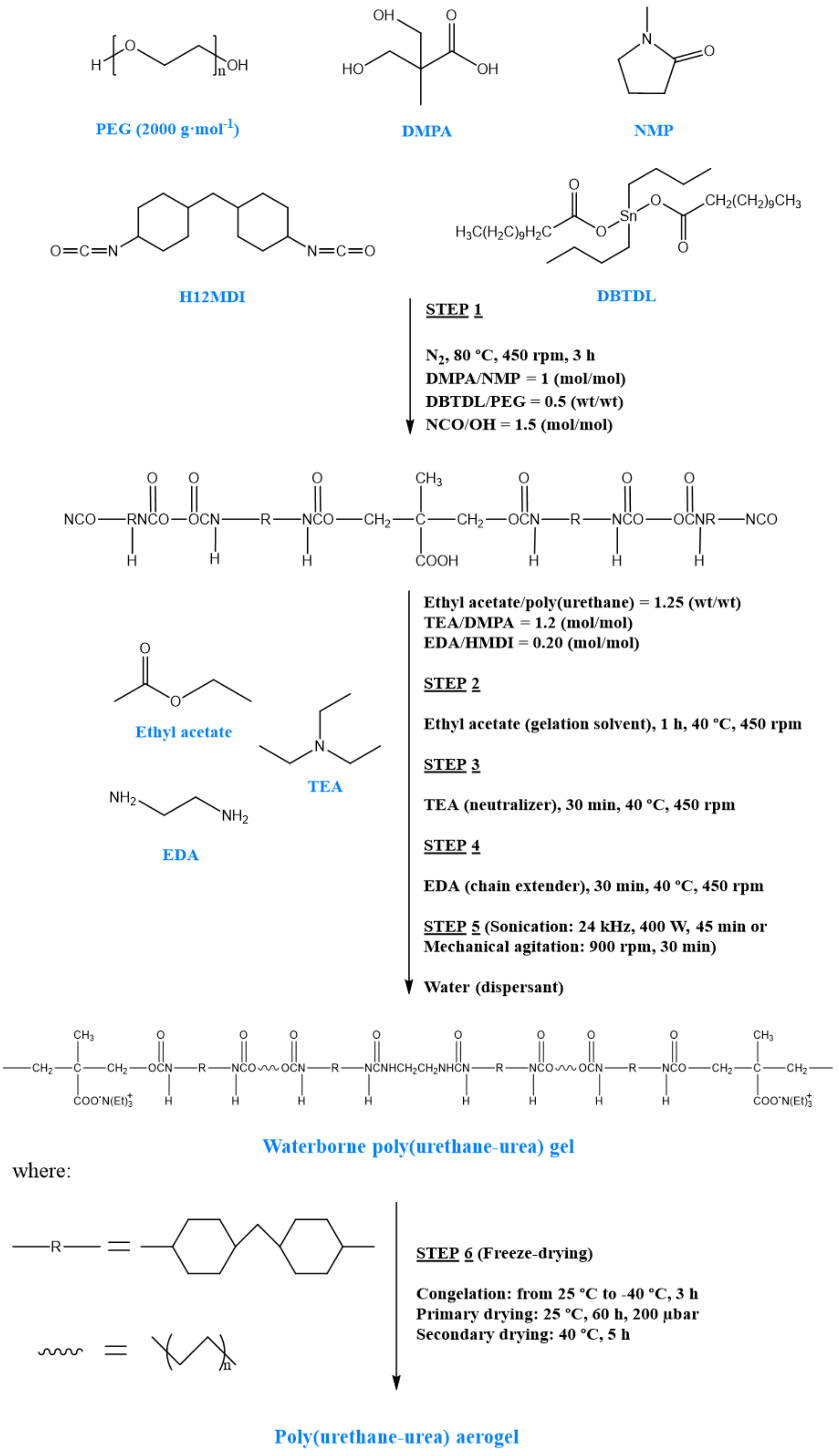

Figure S5 Schematic process of WBPUR aerogel synthesis.

Table S1. Hansen solubility parameters of the organic solvents evaluated.

Solvent 1	Proportion	Solvent 2	Proportion	T^B (ºC)	$\delta_{Dispersion}$ [$MPa^{1/2}$]	$\delta_{Polarity}$ [$MPa^{1/2}$]	$\delta_{H\text{-}bonding}$ [$MPa^{1/2}$]
Acetone	100	-	-	56	15.5	10.4	7.0
ACN	100	EtOAc	-	82	15.3	18.1	6.1
	75		25	-	15.4	14.8	6.4
	50		50	-	15.6	11.7	6.7
	25		75	-	15.7	8.5	6.9
	-		100	77	15.8	5.3	7.2

T^B: Temperature boiling

Hansen solubility parameters of the binary solvent combinations were determined as a function of the two selected solvents in their mixing ratios.
